# Effects of Aerobic and Interval Training on Peripheral Nerve Recovery in Male and Female Diabetic Rats

**DOI:** 10.7759/cureus.87793

**Published:** 2025-07-12

**Authors:** Toru Tamaki, Ken Muramatsu, Masako Ikutomo

**Affiliations:** 1 Department of Physical Therapy, Nagoya Aoi University, Nagoya, JPN; 2 Department of Physical Therapy, Kyorin University, Mitaka, JPN; 3 Department of Physical Therapy, University of Tokyo Health Sciences, Tama, JPN

**Keywords:** diabetes, exercise, motor neuron, peripheral nerve injury, sex differences

## Abstract

Background

Diabetes mellitus negatively affects the peripheral nervous system, often leading to diabetic neuropathy (DN), which impairs nerve regeneration. Although exercise therapy promotes peripheral nerve regeneration, its effects under diabetic conditions remain unclear. The aim of this study was to investigate the effects of aerobic exercise and interval training on nerve regeneration following tibial nerve crush injury in male and female type 1 diabetic and control rats.

Methods

Type 1 diabetes mellitus was induced in Wistar rats via intraperitoneal injection of streptozotocin (50 mg/kg), and diabetes was confirmed after two weeks (blood glucose >400 mg/dL). Diabetic (DM) and age-matched control (CO) rats underwent tibial nerve crush injury and were randomly assigned to sedentary (SED), aerobic training (AT), or interval training (IT) groups. The AT group swam for 10 minutes, five times per week, while the IT group performed eight sets of 20-second swimming with 40-second rest intervals while carrying a weight equivalent to 18% of body weight, five times per week. Nerve recovery was assessed using compound muscle action potential (CMAP), nerve conduction velocity (NCV), and retrograde labeling of motor neurons.

Results

CMAP recovery rate and NCV were significantly lower in diabetic rats than in controls. In male rats, only aerobic training improved CMAP recovery rate and NCV, whereas interval training had no beneficial effects. Conversely, in female rats, both AT and IT significantly enhanced functional recovery, indicating a sex-dependent response. Motor neuron counts were lower in diabetic rats. In males, AT increased the number of motor neurons, whereas no significant changes were observed in females.

Conclusion

The effects of exercise on diabetic nerve regeneration are sex-dependent. In male rats, only aerobic exercise was beneficial, whereas interval training impaired recovery. In female rats, both aerobic and interval training promoted functional recovery. These findings highlight the potential of sex-specific exercise interventions for the management of DN.

## Introduction

Diabetes mellitus affects an increasing number of individuals and can cause various complications. The most common complication is diabetic neuropathy (DN), which causes various disorders of the sensory nerves, motor neurons, and central nervous system [1-3]. These dysfunctions are suspected to contribute to the clinical manifestations of diabetes, such as sensory disturbances, reduced muscle function, and impaired balance. In addition to DN, diabetes impairs nerve regeneration owing to insufficient upregulation of neurotrophic factors and their receptors, disrupted axonal transport of cytoskeletal proteins, impaired nerve synthesis, and abnormal macrophage activity [4-6]. As a result, patients with diabetes are more susceptible to posterior nerve compression and strangulation neuropathy, and often experience poor recovery, making it a clinically important issue [6,7].

Exercise therapy is recognized as an effective strategy to promote nerve regeneration, with evidence supporting its role in enhancing axonal elongation, stimulating Schwann cell proliferation, and improving muscle function [8,9]. In recent years, attention has also turned to sex differences in the effects of exercise on nerve recovery. Aerobic exercise has been reported to be more effective in males, while higher-intensity interval training may be more beneficial in females [10].

However, because diabetes places considerable stress on peripheral nerves, exercise intensity must be set with greater caution when diabetes and peripheral nerve injury coexist [11]. Nevertheless, knowledge regarding the effects of exercise therapy under these combined conditions remains limited. In particular, the impact of sex differences on exercise outcomes in diabetic conditions is not well understood, and in clinical practice, sex is rarely considered when prescribing exercise therapy for patients with diabetes.

Therefore, we hypothesized that the optimal exercise intensity for promoting nerve recovery under diabetic conditions differs between male and female rats. We aimed to explore the relationship between exercise intensity and sex differences, and to identify new possibilities for exercise therapy for peripheral nerve injury under diabetic conditions. To this end, we examined the effects of aerobic and interval training on peripheral nerve regeneration following tibial nerve crush injury in a rat model of type 1 diabetes.

## Materials and methods

General protocol

All experimental procedures were approved by the Animal Ethics Committee of Nagoya Aoi University (Nagoya, Japan, approval No: 2022-2). All animal experiments were conducted in accordance with the National Institutes of Health Guide for the Care and Use of Laboratory Animals (NIH Publication No. 8023; revised 1978). Anesthesia was induced and maintained via inhalation of 5% and 3.0-3.5% isoflurane, respectively. Rectal temperature was maintained at 37-38°C using a heating blanket. 

Induction of experimental diabetes 

Type 1 diabetes was induced in 13-week-old male (n=15) and female (n=15) Wistar rats by intraperitoneal injection of streptozotocin (STZ, 50 mg/kg in saline). Two weeks after STZ injection, random plasma glucose levels were measured from tail vein blood using a portable glucose meter (Accu-Chek Aviva Nano; Roche Diagnostics, Basel, Switzerland). Animals with glucose levels exceeding 400 mg/dL were defined as diabetic. Age-matched control animals (15 male and 15 female rats) received saline injections only. All animals were housed in flat-bottomed plastic cages containing soft bedding material. Food and tap water were provided ad libitum, and all animals were maintained in a temperature-controlled room with a light-dark cycle of 12:12 h. Male and female rats that received STZ treatment (DM) and control rats (CO) were randomly assigned to three groups: sedentary (SED), aerobic training (AT), and interval training (IT) groups. Each of the SED (DM-SED, CO-SED), AT (DM-AT, CO-AT), and IT (DM-IT, CO-IT) groups comprised five rats per subgroup (DM and CO).

**Figure 1:**
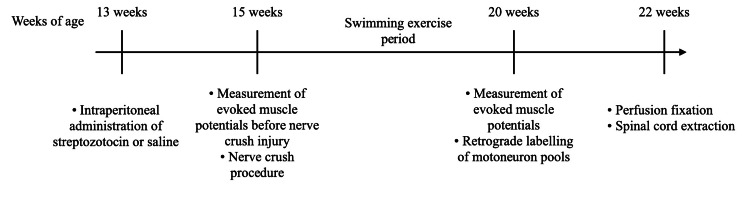
Experimental design Timeline of the experimental procedures from 13 to 22 weeks of age. Image Credits: Authors

The study design is illustrated in Figure 1. All rats underwent electrical stimulation of the sciatic nerve to measure the compound muscle action potential (CMAP) of the medial gastrocnemius (MG) muscle, followed by tibial nerve crush injury. Three days post-injury, a five-week exercise period was initiated, after which CMAP measurements were repeated. Subsequently, a fluorescent neurotracer was injected into the MG to label lumbar motor neurons, followed by a two-week survival period. Finally, perfusion fixation was performed, and the spinal cord was extracted.

Recording of evoked electromyography potential

The measurement of evoked electromyography (EMG) was performed under anesthesia. The sciatic nerve was exposed at the sciatic rectal fossa and secured using a bipolar silver hook electrode for stimulation. A single supramaximal pulse was applied at 1 s intervals (pulse duration 0.1 ms, intensity 3-5 V). The CMAP of the MG was recorded using a bipolar silver ball electrode pressed against the center of the muscle belly. Data were digitized at a sampling rate of 200 kHz using PowerLab (AD Instruments). From 30 recorded measurements, the maximal peak-to-peak amplitude of the evoked CMAP and nerve conduction velocity (NCV) were analyzed. NCV was calculated by dividing the distance between the stimulation and recording electrodes by the latency. Subsequently, the tibial nerve was crushed using forceps for 30 s at a proximal position, 3 mm from its insertion into the muscle. After the crush injury, CMAP was measured again using the same method, confirming that the amplitude had decreased to <5% of the preinjury value. Following the exercise period, CMAP was remeasured using the same procedure. The obtained values were compared with pre-injury values and expressed as percentages.

Retrograde labeling of motoneuron pools

All animals were anesthetized with 5% isoflurane for induction and subsequently maintained under 3.0-3.5% isoflurane inhalation. Fluororuby (FR, Dextran tetramethylrhodamine, Molecular Probes, D-1817) was injected into the MG to label its motor neurons retrogradely. Approximately 25 µL of the tracer was evenly injected into the MG muscle using a micro-syringe. The syringe was carefully withdrawn to prevent leakage, the injection site was cleaned, and the skin was sutured. Fourteen days later, the animals were re-anesthetized in a chamber filled with isoflurane and perfused transcardially with 500-1000 mL normal saline containing heparin sodium, followed by 4% paraformaldehyde in 0.1 mol/L phosphate buffer (pH 7.4) at 4°C. The spinal level was identified using the nerve roots as anatomical landmarks, and the L2-S3 portion of the spinal cord was then dissected and post-fixed in cold fixative (4% paraformaldehyde in 0.1 mol/L phosphate buffer (pH 7.4, 4°C)) for 24 h. Next, longitudinal sections (80 μm thick) of the L2-S3 spinal cord were prepared using a vibratome. These sections were mounted on glass slides, examined, and photographed using a multifocal fluorescence microscope (BZ-X710, KEYENCE, Osaka, Japan). Labeled motor neurons were observed using objective lenses with 20×40 magnification and a fluorescence filter set with an excitation wavelength of 540-560 nm and an emission wavelength of 575-620 nm. The largest cross-sectional area of motor neuron cell bodies and the total number of motor neurons were calculated using the ImageJ software. For area measurements, the inner boundaries of cell bodies containing clearly identifiable nuclei were manually traced. Cells with indistinct borders were excluded from the analysis. Cell bodies fragmented across multiple images were reconstructed using Photoshop CS6 (Adobe, San Jose, CA, USA) and analyzed. Motor neurons were excluded if reconstruction was not feasible. 

Exercise protocol

Swimming exercise was initiated three days after the nerve crush injury. The exercise was conducted in a cylindrical container with a water depth of 40 cm, maintained at a temperature of 30-35°C. The AT group performed continuous aerobic swimming for 10 minutes per session, five days per week, based on a previous study reporting its effectiveness in promoting nerve regeneration [12]. The IT group performed high-intensity interval swimming with weights equivalent to 18% of body weight. Each session consisted of eight sets of 20 seconds of swimming followed by 40 seconds of rest, conducted five days per week, as described in a previous study [13].

Data analysis

Data are presented as mean ± SD. Owing to the limited sample size, group comparisons were primarily conducted using two-way analysis of variance (ANOVA), followed by Tukey’s multiple-comparison test to identify specific group differences. Paired-sample t-tests were used to compare blood glucose levels before and after the exercise period.

In addition, an exploratory three-way ANOVA was performed to assess the main effects and potential interactions of diabetes status (diabetic vs. control), sex (male vs. female), and exercise type (sedentary, aerobic, or interval training) on the measured variables. All analyses were performed using Prism, version 7 (GraphPad Software, La Jolla, CA, USA). Statistical significance was defined as P<0.05, with a trend toward significance defined as 0.05≤P<0.15.

## Results

Body weight and blood glucose levels

The body weights and blood glucose levels of the rats in all groups are summarized in Table 1. The body weight of diabetic rats was significantly lower than that of control rats for both males and females. Additionally, in male rats, five weeks after nerve crush injury, the control group showed a trend toward lower body weight in the AT and IT groups, compared with the SED group (AT: P=0.09, IT: P=0.07). In contrast, in diabetic rats, the AT and IT groups tended to have higher body weights than the SED group (AT: P=0.11, IT: P=0.07). In female rats, no significant exercise-induced changes in body weight were observed.

**Table 1:** Body weight and blood glucose level ^a^P<0.05 vs. CO-SED Data presented as mean ± SD unless otherwise indicated. STZ, streptozotocin; CO-SED, control-sedentary group; CO-AT, control-aerobic training group; CO-IT, control-interval training group; DM-SED, diabetic-sedentary group; DM-AT, diabetic-aerobic training group; DM-IT, diabetic-interval training group

	Before nerve crush (2 weeks after STZ or saline administration)												5 weeks after nerve crush (7 weeks after STZ or saline administration)											
	CO-SED (n=5)		CO-AT (n=5)		CO-IT (n=5)		DM-SED (n=5)		DM-AT (n=5)		DM-IT (n=5)		CO-SED (n=5)		CO-AT (n=5)		CO-IT (n=5)		DM-SED (n=5)		DM-AT (n=5)		DM-IT (n=5)	
Male																								
Body weight (g)	315.2±3.48		315.8±5.63		312.2±2.03		227.0±9.57^a^		229.2±11.72^a^		237.6±12.05^a^		356.6±9.64		339.8±3.37		339.0±6.69		192.0±5.21^a^		208.0±10.15^a^		209.4±12.35^a^	
Blood glucose (mg/dL)	127.0±8.02		134.6±7.55		127.0±9.57		539.4±29.38^a^		502.8±66.76^a^		516.4±56.92^a^		127.6±8.42		136.2±5.67		127.0±6.41		519.2±68.22^a^		463.0±36.98^a^		469.4±29.97^a^	
Female																								
Body weight (g)	202.6±4.96		203.2±2.78		200.8±3.86		173.2±9.10^a^		172.6±3.32^a^		176.2±4.35^a^		211.8±5.84		215.2±5.19		211.4±4.49		160.0±7.15^a^		157.4±5.60^a^		160.6±5.16^a^	
Blood glucose (mg/dL)	133.2±8.10		132.0±3.57		129.0±6.41		496.8±33.10^a^		513.4±51.67^a^		531.6±61.83^a^		130.0±9.52		130.2±6.30		130.4±8.54		497.6±32.05^a^		471.2±37.03^a^		511.2±51.88^a^	

**Figure 2:**
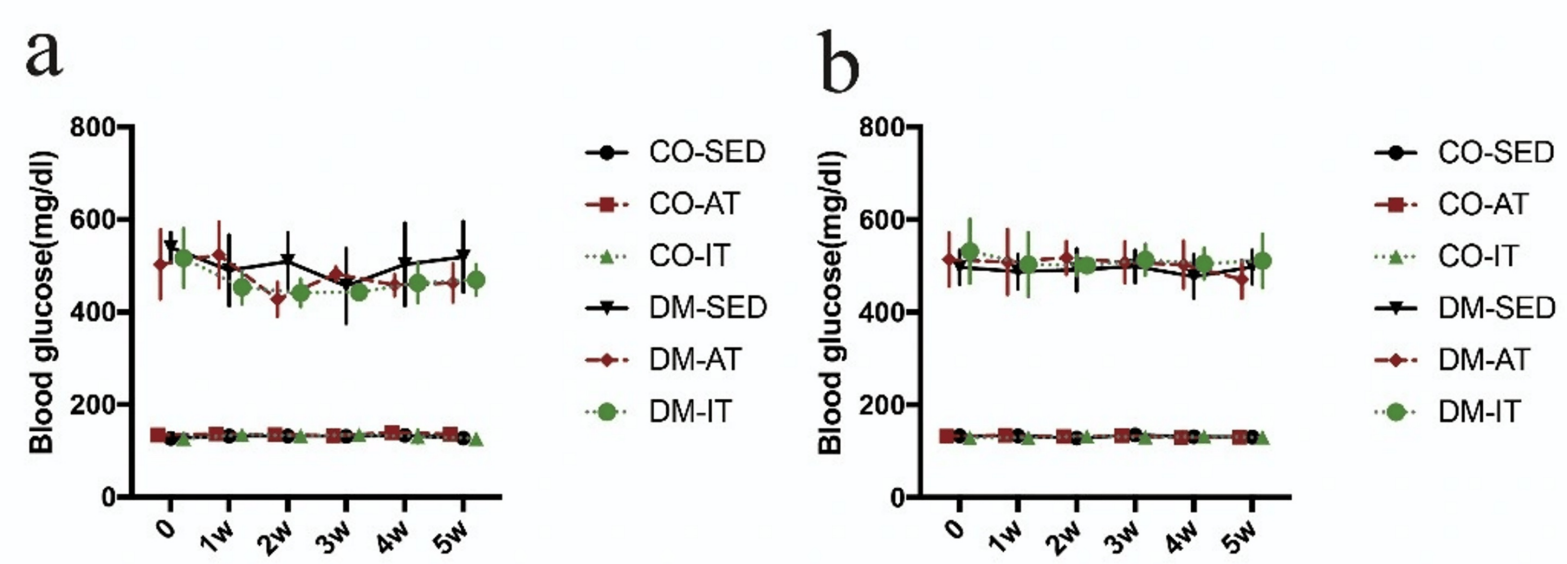
Alterations in blood glucose levels during the exercise period (a) Blood glucose levels in male rats and (b) blood glucose levels in female rats. No significant changes in blood glucose levels were observed during the exercise period. CO-SED, control-sedentary group; CO-AT, control-aerobic training group; CO-IT, control-interval training group; DM-SED, diabetic-sedentary group; DM-AT, diabetic-aerobic training group; DM-IT, diabetic-interval training group

Blood glucose levels were higher in the DM group than in the CO group. However, no significant changes in blood glucose levels were observed between the pre-crush and five weeks post-crush periods. Figure 2 shows changes in blood glucose levels over time in each group.

Electrophysiological function and muscle weight

**Figure 3:**
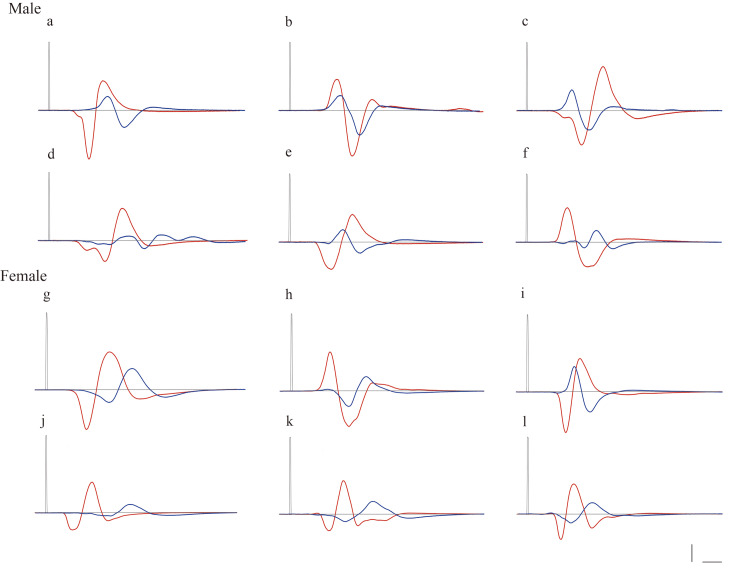
Waveform of CMAP (a-f) CMAPs in male rats: (a) CO-SED, (b) CO-AT, (c) CO-IT, (d) DM-SED, (e) DM-AT, and (f) DM-IT. (g-l) CMAPs in female rats: (g) CO-SED, (h) CO-AT, (i) CO-IT, (j) DM-SED, (k) DM-AT, (l) DM-IT. The vertical scale bar indicates 1.0 V and the horizontal scale bar corresponds to 100 ms. The red line represents CMAPs before nerve crush injury, and the blue line represents CMAPs after the exercise period (five weeks post-crush). CMAP, compound muscle action potential; CO-SED, control-sedentary group; CO-AT, control-aerobic training group; CO-IT, control-interval training group; DM-SED, diabetic-sedentary group; DM-AT, diabetic-aerobic training group; DM-IT, diabetic-interval training group

Figure 3 presents the representative CMAP waveforms, and Table 2 summarizes the percentages of post-exercise CMAP peak-to-peak amplitude relative to pre-crush values, NCV after the exercise period, and muscle weight.

**Table 2:** CMAP and muscle weight ^a^P<0.05 vs. CO-SED ^b^P<0.05 vs. DM-SED Data presented as mean ± SD unless otherwise indicated. CMAP, compound muscle action potential; CO-SED, control-sedentary group; CO-AT, control-aerobic training group; CO-IT, control-interval training group; DM-SED, diabetic-sedentary group; DM-AT, diabetic-aerobic training group; DM-IT, diabetic-interval training group; NCV, nerve conduction velocity

	CO-SED (n=5)		CO-AT (n=5)		CO-IT (n=5)		DM-SED (n=5)		DM-AT (n=5)		DM-IT (n=5)	
Male												
% CMAP peak-to-peak amplitude	37.55±2.17^b^		44.93±3.76^a,b^		54.65±2.95^a,b^		22.87±3.18^a^		31.72±1.60^b^		27.05±4.75^a^	
NCV (ms)	38.63±3.31^b^		46.10±2.14^a,b^		46.93±1.72^a,b^		29.65±1.24^a^		37.61±3.32^b^		28.96±6.78^a,b^	
Muscle weight (g)	0.55±0.03^b^		0.57±0.02^b^		0.62±0.01^a,b^		0.31±0.02^a^		0.31±0.01^a^		0.33±0.01^a^	
Female												
% CMAP peak-to-peak amplitude	40.43±2.59^b^		40.99±3.51^b^		62.55±8.84^a,^^b^		23.96±3.52^a^		32.90±3.33		34.53±3.18^b^	
NCV (ms)	39.1±1.58^b^		39.76±3.62^b^		54.22±3.28^a,b^		27.4±1.83^a^		36.1±2.55^b^		35.56±5.93^b^	
Muscle weight (g)	0.34±0.01^b^		0.34±0.01^b^		0.39±0.01^a,b^		0.21±0.01^a^		0.21±0.01^a^		0.24±0.01^a^	

**Figure 4:**
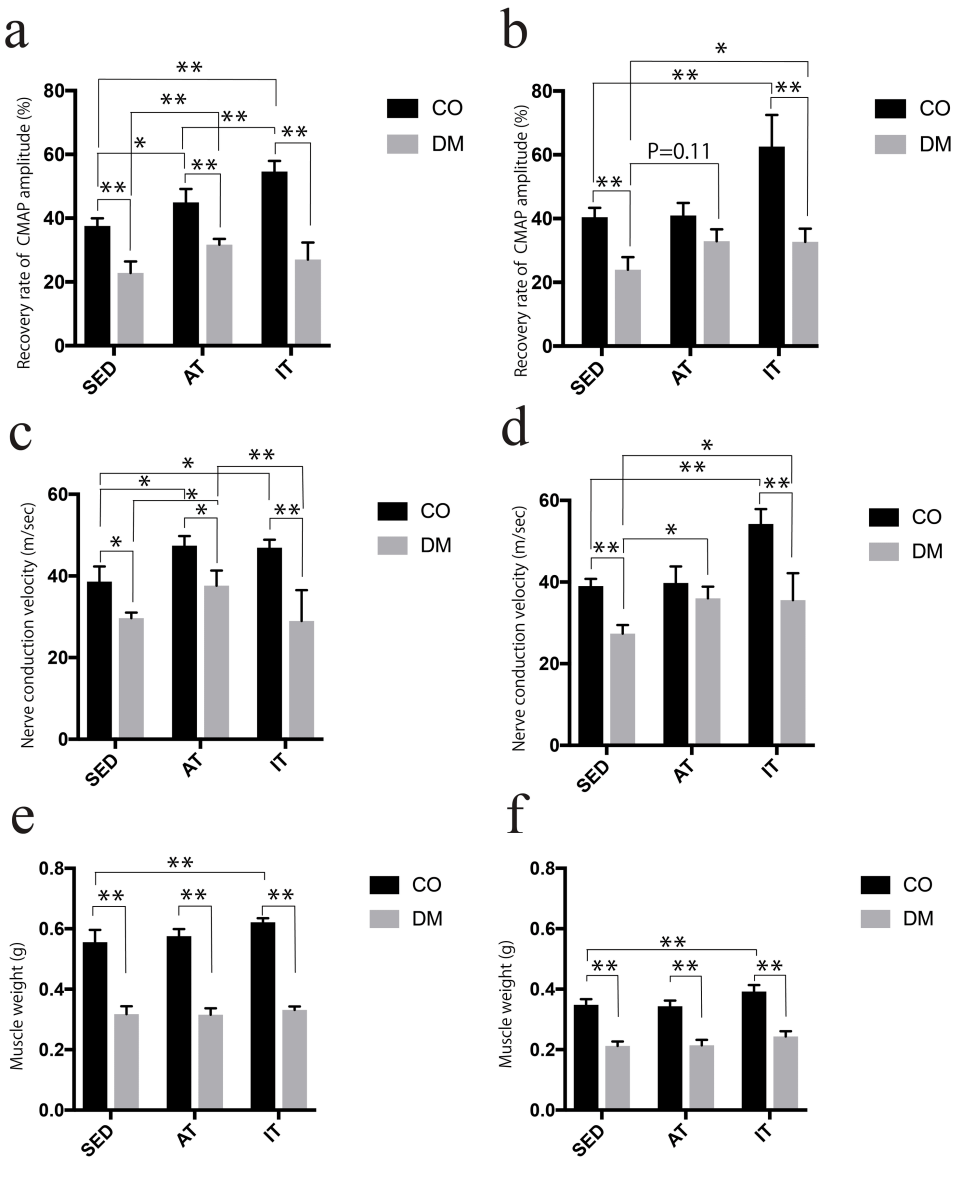
Recovery rate of CMAP and muscle weight *P<0.05 **P<0.01 CMAP amplitude recovery rate in (a) male and (b) female rats. NCV in (c) male and (d) female rats. Muscle weight of (e) male and (f) female rats. CMAP, compound muscle action potential; CO, control group; DM, diabetic group; SED, sedentary group; AT, aerobic training group; IT, interval training group; NCV, nerve conduction velocity

The amplitude recovery rate was lower in diabetic rats than in controls for both sexes. Control male rats showed the highest values in the order of SED<AT<IT. Diabetic rats showed higher values in the AT group than in the SED group, with no significant difference between the IT and SED groups. Female control rats showed a significantly higher value in the IT group than in the SED group. Diabetic rats showed higher values in both the AT and IT groups than in the SED group (Figures 4a, 4b). In the three-way ANOVA, significant main effects were observed for diabetes status (F (1,48)=240.60, P<0.001), sex (F (1,48)=5.67, P=0.021), and exercise type (F (2,48)=45.05, P<0.001). Significant interaction effects were observed between diabetes status and exercise type (F (2,48)=19.31, P<0.001) as well as between sex and exercise type (F (2,48)=5.20, P=0.009). However, the interaction between diabetes status and sex (F (1,48)=0.18, P=0.676) and the three-way interaction (F (2,48)=0.83, P=0.444) were not significant.

NCV was also lower in diabetic rats than in controls for both sexes. Male control rats showed higher values in the AT and IT groups than in the SED group, with no significant difference between the AT and IT groups. Diabetic rats showed higher values in the AT group than in both the SED and IT groups. Female control rats showed higher NCV values in the IT group than in the SED and AT groups. Diabetic rats showed higher NCV values in both the AT and IT groups than in the SED group, with no significant differences between the AT and IT groups (Figures 4c, 4d). In the three-way ANOVA, significant main effects were observed for diabetes status (F (1,48)=129.79, P<0.001) and exercise type (F (2,48)=21.70, P<0.001). Significant interaction effects were also found between diabetes status and exercise type (F (2,48)=12.40, P<0.001), as well as between sex and exercise type (F (2,48)=10.26, P=0.0002). In contrast, no significant main effect of sex (F (1,48)=0.44, P=0.511), no significant interaction between diabetes status and sex (F (1,48)=0.06, P=0.816), and no significant three-way interaction (F (2,48)=1.17, P=0.319) were observed.

Muscle weight was lower in both male and female diabetic rats than in controls. Control rats had a significant increase in muscle weight in the IT group. However, no significant differences were observed among the three groups in diabetic rats (Figures 4e, 4f). In the three-way ANOVA, significant main effects were observed for diabetes status (F (1,48) = 1301.63, P<0.001), sex (F (1,48)=840.68, P<0.001), and exercise type (F (2,48)=19.91, P<0.001). Significant interaction effects were also found between diabetes status and sex (F (1,48)=126.01, P<0.001). In contrast, a trend toward an interaction between diabetes status and exercise type was observed (F (2,48)=3.00, P=0.059), although it did not reach significance. Neither significant interaction was found between sex and exercise type (F (2,48)=0.30, P=0.740), nor was there a significant three-way interaction (F (2,48)=1.14, P=0.329).

Morphological alterations of motor neurons

**Figure 5:**
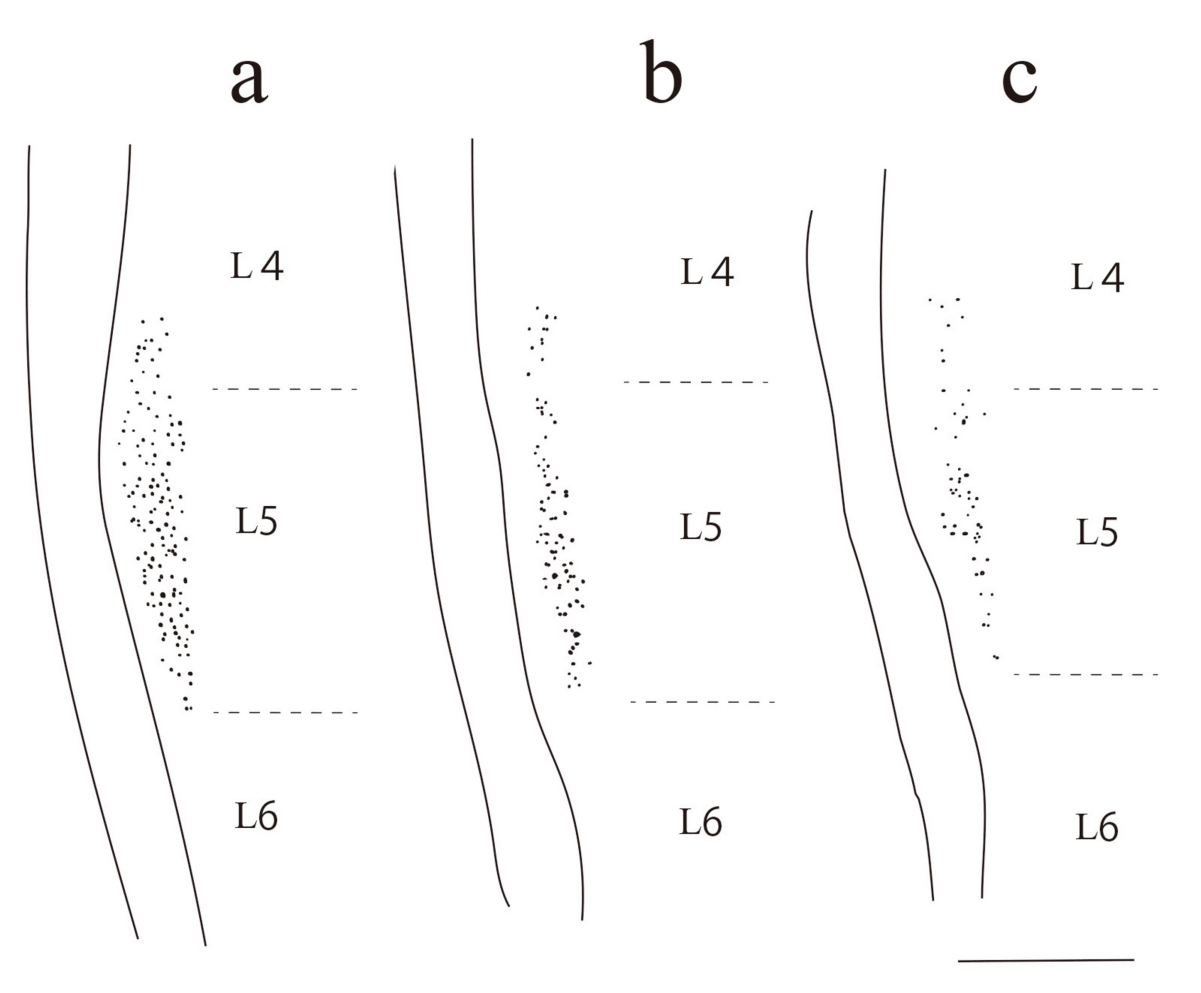
Reconstruction of MG nuclei Reconstruction of MG nuclei in male rats: (a) intact CO-SED, (b) CO-SED, and (c) DM-SED. The image shows an overlay view of all labeled MG motoneuron cell body contours from a representative single animal. The zone between the bold lines represents the white matter, and horizontal solid lines indicate the boundaries of the L4, L5, and L6 spinal segments. The scale bar represents 1 mm. MG, medial gastrocnemius; CO-SED, control-sedentary group; DM-SED, diabetic-sedentary group Image Credits: Authors

**Figure 6:**
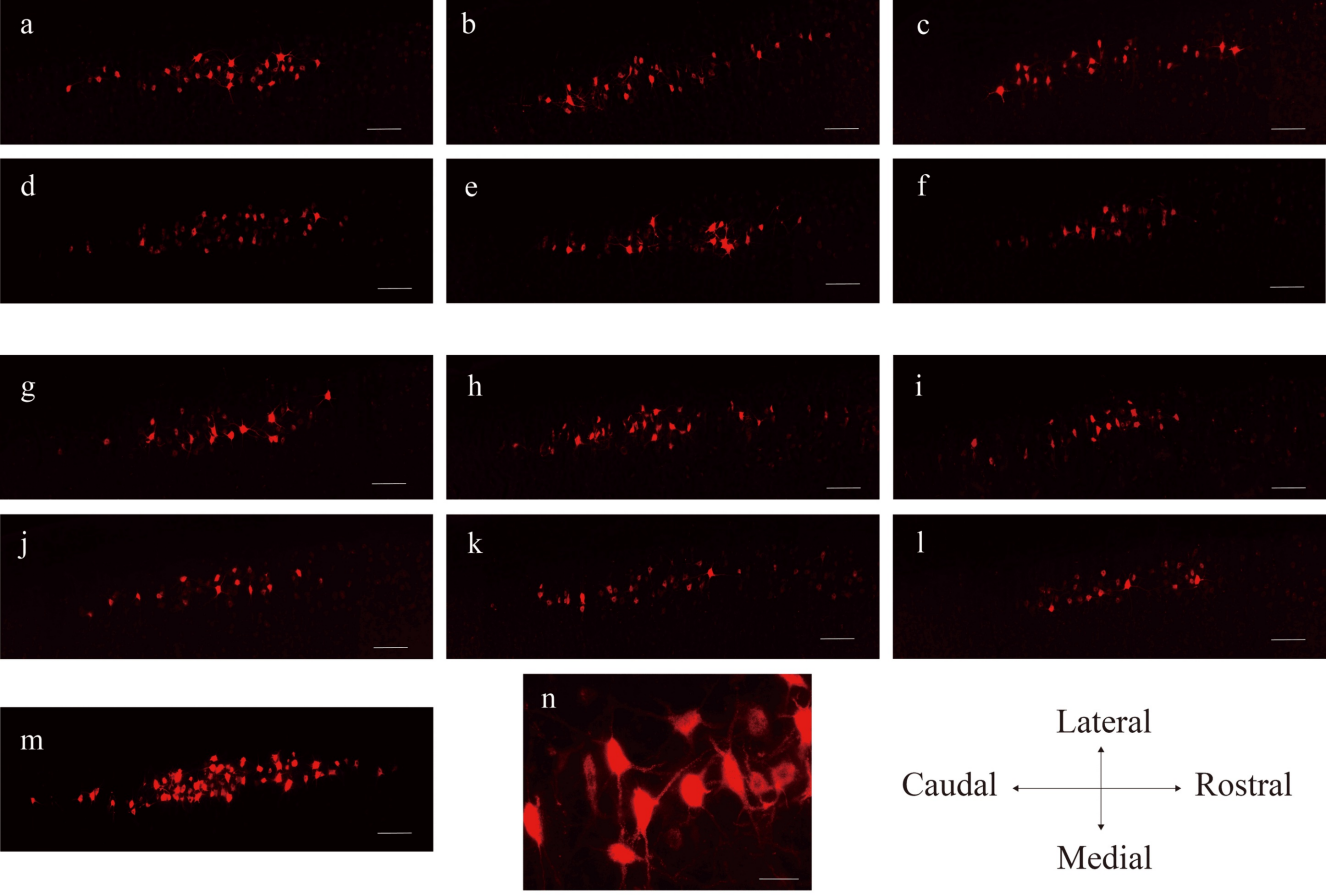
Representative images of MG cell columns in a single plane Representative images of MG cell columns in a single plane with the largest number of motor neurons. (a-f) MG motor columns in male rats: (a) CO-SED, (b) CO-AT, (c) CO-IT, (d) DM-SED, (e) DM-AT, and (f) DM-IT. (g-l) MG motor columns in female rats: (g) CO-SED, (h) CO-AT, (i) CO-IT, (j) DM-SED, (k) DM-AT, and (l) DM-IT. (m) MG motor columns in the non-crushed tibial nerve of the male control group. Scale bars represent 200 μm. (n) High-magnification images of the cell bodies of motor neurons. The scale bar indicates a measurement of 50 μm. MG, medial gastrocnemius; CO-SED, control-sedentary group; CO-AT, control-aerobic training group; CO-IT, control-interval training group; DM-SED, diabetic-sedentary group; DM-AT, diabetic-aerobic training group; DM-IT, diabetic-interval training group

All retrogradely labeled motor neurons were localized in the ventral horn of the spinal cord. They were arranged in a longitudinal cell column and predominantly located between the L4 and L5 spinal segments (Figure 5). Figures 6a-6f depict representative MG motor neuron columns for each group in male rats, whereas Figures 6g-6l illustrate those in female rats. All motor neurons were identified based on their cell bodies and dendrites.

Figure 6m presents an image of the motor neuron column in control male rats, showing the contralateral, non-crushed motor neurons of the MG. The average number of motor neurons was 116 (N=3), which is comparable to that reported in previous studies [14]. A high-magnification image is presented in Figure 6n. The number and cross-sectional area of MG motor neurons are summarized in Table 3.

**Table 3:** Number and cross-sectional area of labeled motor neurons ^a^P<0.05 vs. CO-SED Data presented as mean ± SD unless otherwise indicated. CO-SED, control-sedentary group; CO-AT, control-aerobic training group; CO-IT, control-interval training group; DM-SED, diabetic-sedentary group; DM-AT, diabetic-aerobic training group; DM-IT, diabetic-interval training group

	CO-SED (n=5)		CO-AT (n=5)		CO-IT (n=5)		DM-SED (n=5)		DM-AT (n=5)		DM-IT (n=5)	
Male	n=407 cells; from 5 animals		n=507 cells; from 5 animals		n=427 cells; from 5 animals		n=331 cells; from 5 animals		n=395 cells; from 5 animals		n=340 cells; from 5 animals	
Cross-sectional area (μm^2^)	722.49±60.41		735.03±36.80		743.08±38.70		663.21±56.21		721.79±10.64		754.34±32.70	
Number of motor neurons	81.4±8.4		101.4±9.1^a^		84.8±10.9		66.2±4.4		79.0±9.0		68.0±7.4	
Female	n=416 cells; from 5 animals		n=465 cells; from 5 animals		n=458 cells; from 5 animals		n=340 cells; from 5 animals		n=365 cells; from 5 animals		n=375 cells; from 5 animals	
Cross-sectional area (μm^2^)	682.88±21.49		674.10±30.98		700.66±34.86		703.20±25.38		688.90±7.11		652.17±18.71	
Number of motor neurons	83.2±8.9		93.0±9.0		91.6±7.3		68.0±6.8		73.0±8.1		75.0±8.4	

**Figure 7:**
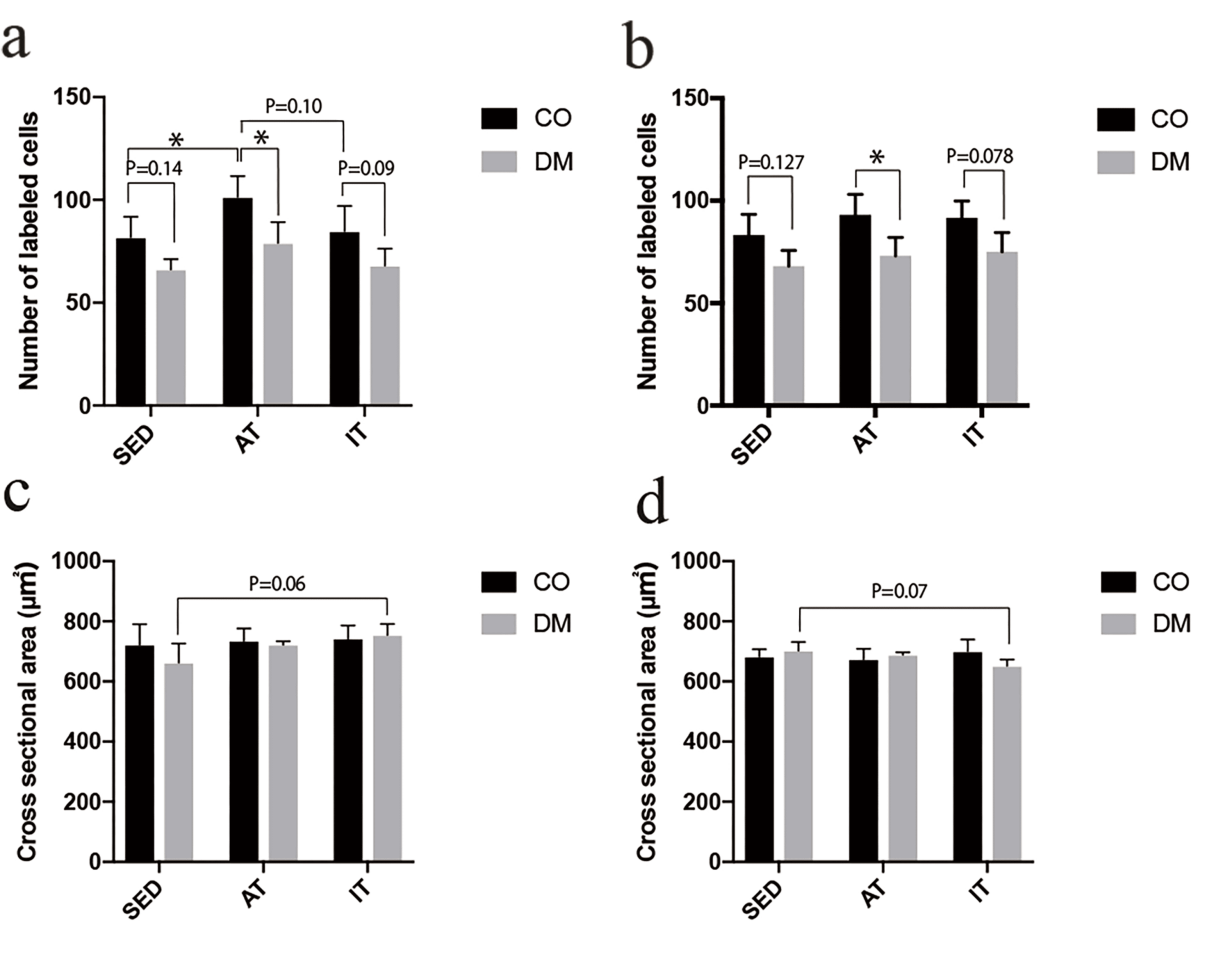
Number of labeled MG motor neurons and cross-sectional area *P<0.05 Number of labeled MG motor neurons in (a) male and (b) female rats. Cross-sectional area of labeled MG motor neurons in (c) male and (d) female rats. MG, medial gastrocnemius; CO, control group; DM, diabetic group; SED, sedentary group; AT, aerobic training group; IT, interval training group

The number of motor neurons was lower in diabetic rats than in controls for both sexes. In male control rats, a significant increase was observed in the AT group compared with the SED group, whereas the value decreased in the IT group compared with the AT group. Contrastingly, in diabetic rats, the AT group showed an increase of approximately 19% compared with the SED group and approximately 16% compared with the IT group; however, these differences were not statistically significant (Figure 7a). Female control rats showed an increase of approximately 11% in the AT group and 10% in the IT group compared with the SED group, although these differences were not statistically significant. Similarly, diabetic rats showed an increase of approximately 7% in the AT group and 10% in the IT group compared with the SED group, with no statistically significant differences (Figure 7b). In the three-way ANOVA, significant main effects were observed for diabetes status (F (1,48)=54.17, P<0.001) and exercise type (F (2,48)=8.21, P<0.001), indicating that both diabetes and exercise independently influenced the count. No significant main effect of sex (F (1,48)=0.043, P=0.836) was found. Additionally, interaction effects were not significant between diabetes status and sex (F (1,48)=0.032, P=0.858), between diabetes status and exercise type (F (2,48)=0.562, P=0.574), or among all three factors (F (2,48)=0.026, P=0.975). A trend toward an interaction between sex and exercise type was observed (F (2,48)=2.94, P=0.063), although it did not reach significance.

The cross-sectional area of motor neurons did not show significant differences among groups in either male or female rats. However, in male rats, the DM-IT group tended to have a larger cross-sectional area than the DM-SED group. Contrastingly, female rats in the DM-IT group tended to have smaller cross-sectional areas than those in the DM-SED group (Figures 7c, 7d). In the three-way ANOVA, a significant main effect of sex was observed (F (1,48)=15.60, P=0.00026). In contrast, no significant main effects were found for diabetes status (F (1,48)=1.53, P=0.222) or exercise type (F (2,48)=1.29, P=0.284). Significant interaction effects were found between sex and exercise type (F (2,48)=4.47, P=0.017) and for the three-way interaction among diabetes status, sex, and exercise type (F (2,48)=4.10, P=0.023). In contrast, the interaction between diabetes status and sex (F (1,48)=0.63, P=0.431) and the interaction between diabetes status and exercise type (F (2,48)=0.43, P=0.651) were not significant.

**Figure 8:**
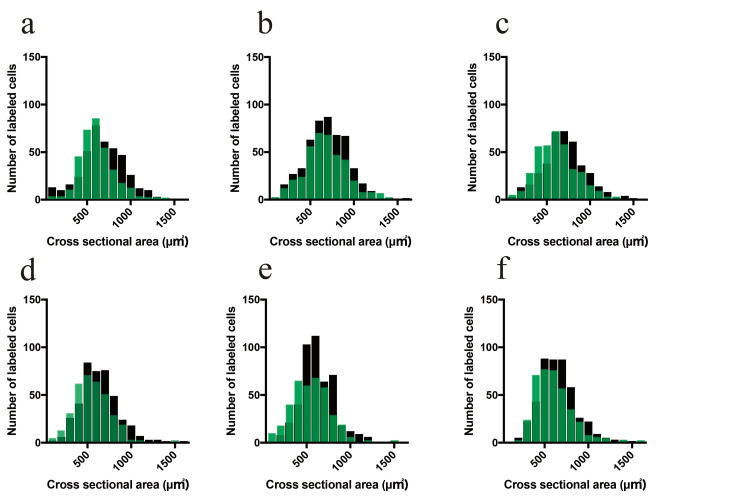
Size distribution of MG motor neurons Frequency histograms of the cross-sectional area of motor neuron cell bodies. Black bars represent the control (CO) group, and green bars represent the diabetic (DM) group. (a-c) Histogram of MG motor neurons in male rats: (a) CO-SED, DM-SED; (b) CO-AT, DM-AT; (c) CO-IT, DM-IT. (d-f) Histograms of MG motor neurons in female rats: (d) CO-SED, DM-SED; (e) CO-AT, DM-AT; (f) CO-IT, DM-IT. MG, medial gastrocnemius; CO-SED, control-sedentary group; CO-AT, control-aerobic training group; CO-IT, control-interval training group; DM-SED, diabetic-sedentary group; DM-AT, diabetic-aerobic training group; DM-IT, diabetic-interval training group

Figure 8 presents histograms of the cross-sectional area of motor neuron cell bodies in each experimental group. In male rats, both the control and diabetic groups showed a unimodal distribution with a peak at approximately 600 μm². Additionally, in diabetic rats, a tendency for a reduction in small-sized motor neurons (<400 μm²) was observed. In female rats, a unimodal distribution was also observed, peaking at approximately 600 μm². However, in the diabetic group, there was a tendency for a reduction in motor neurons ranging from 600 to 700 μm².

## Discussion

The findings of this study suggest two key implications. First, functional recovery following peripheral nerve injury is impaired by the presence of diabetes. Second, exercise facilitates nerve recovery, but the effectiveness and tolerance of exercise may differ between sexes. 

Diabetes has been shown to negatively affect nerve regeneration through several mechanisms, including oxidative stress and ischemia induced by hyperglycemia, resulting in reduced expression of neurotrophic factors, and impaired synthesis of neurofilaments and tubulin. Furthermore, diabetes has also been reported to delay the initiation of nerve regeneration [15-17]. These factors likely contributed to the observed reductions in CMAP recovery rate, NCV, and the number of motor neurons in the diabetic groups. 

Sex differences were observed in the effects of exercise. In the control group, the CMAP recovery rate was increased in male rats in the order of AT followed by IT, while in female rats, an increase was observed only in the IT group. Similarly, NCV was increased in both the AT and IT groups in male rats and in the IT group in female rats, suggesting that axonal conduction function may have improved. Previous studies have shown that low-intensity treadmill exercise promotes axonal regeneration in males, and interval training does so in females, supporting the findings of the present study [10]. However, previous studies have not reported axonal regeneration in male rats following interval training. In this regard, it is important to note that CMAP recovery reflects not only improvements in axonal transmission and reinnervation but also changes in muscle properties, including hypertrophy. In the present study, increased muscle mass was observed in the CO-IT group, indicating that the observed CMAP recovery in this group may have been partially attributed to muscle hypertrophy in addition to axonal regeneration. Additionally, the difference in recovery outcomes may be influenced by the type of nerve injury model used, since previous studies have reported variations in recovery depending on whether the injury involved nerve transection or crush injury [18].

In the diabetic group, CMAP recovery and NCV were increased in the male AT group, whereas lower values were observed in the IT group than in the AT group. Unlike the control group, in which both the AT and IT groups showed exercise effects, the effect of IT exercise was not observed in the diabetic group. This may be owing to the combined stress on the nerves from hyperglycemia and high-intensity exercise, which may have counteracted the beneficial effects of exercise. Interestingly, in female rats, both the AT and IT groups showed comparable improvements, suggesting that females may have higher tolerance to high-intensity exercise than males. Although the present study could not clarify the mechanisms underlying these sex differences, several factors may be involved, including the role of androgens in axonal regeneration, differences in glucose and lipid metabolism during exercise, and varying sympathetic nervous system responses [19-22].

A significant increase in the number of motor neurons was observed in the CO-AT group compared with the CO-SED group in male rats. Furthermore, histogram analysis showed a tendency toward neuronal shrinkage and reduction in the number of small-sized motor neurons in the diabetic group, which was consistent with previous reports indicating that small gamma motor neurons are particularly vulnerable to diabetes [23]. The tracer used in this study was taken up by endocytosis and transported retrogradely; therefore, the difference in labeling numbers may reflect the number of motor neurons with remaining axonal damage sufficient to impair tracer transport [24]. However, the difference in number of motor neurons between the control and diabetic groups was relatively minor, compared with the changes in CMAP amplitude recovery rate and NCV, and the effects of exercise were scarcely observed, particularly in the diabetic group. Previous studies have reported that, in diabetes, early-stage impairments such as reduced NCV and neuromuscular junction dysfunction occur before the actual loss of motor neurons [25]. Therefore, the results suggest that exercise therapy is more effective against milder impairments such as decreased NCV than irreversible damage such as motor neuron loss, and that its effectiveness is influenced by both sex and exercise intensity. These findings provide fundamental evidence for the need to tailor exercise intensity based on sex when designing exercise interventions for diabetic patients.

The relationship between exercise and functional recovery is not fully understood because peripheral nerve injury and diabetes have a complex interaction. Exercise promotes nerve regeneration by increasing the expression of neurotrophic factors [26]. Additionally, exercise therapy for diabetes improves glucose metabolism, reduces oxidative stress, and enhances blood flow, which counteracts the negative effects of hyperglycemia [27,28]. Furthermore, even low-intensity exercise, which does not significantly improve blood glucose levels, has been reported to be beneficial for muscle function and motor neuron integrity [14]. In this study, an insulin-deficient type 1 diabetes model induced by STZ was used, and therefore, exercise therapy did not result in significant improvements in blood glucose levels. Therefore, the observed functional recovery was likely due to the release of neurotrophic factors induced by muscle and nerve activity rather than blood glucose control.

This study has some limitations. First, morphological data on nerve axons, muscles, and neuromuscular junctions were not examined. In this study, only motor neuron cell bodies were observed. However, CMAP recovery rate and NCV are likely to be influenced by degeneration of nerve axons and neuromuscular junctions caused by diabetes and peripheral nerve injury. Therefore, a more detailed investigation is needed. Second, although previous studies have suggested that sex hormones and metabolic differences contribute to differential responses, this study did not address the role of these hormones. In addition, the relatively small sample size in each group may have influenced the statistical outcomes related to sex differences and exercise effects. Third, although the exercise protocols were adapted from previous studies, exercise intensity was not determined based on physiological indices, making it impossible to fully isolate the effects of exercise intensity from those of total workload. Future studies should be conducted using a more clinically relevant type 2 diabetes model to further investigate this issue.

## Conclusions

This study demonstrated that the effects of exercise on peripheral nerve injury in diabetes differ by sex, based on the analysis of electrophysiological functions, including CMAP recovery rate and NCV, as well as motor neuron assessment. In male rats, functional recovery was observed only with aerobic exercise, whereas the beneficial effects of exercise were diminished with interval exercise. In contrast, in female rats, both aerobic and interval exercises promoted functional improvements. These findings suggest that male subjects may be more susceptible to stress from high-intensity exercise, highlighting the importance of carefully adjusting exercise intensity under diabetic conditions.

This study contributes to the development of sex-specific exercise strategies for the management of peripheral nerve injuries in patients with diabetes.
